# A Treatise on Disease of the Rectum, Anus and Sigmoid Flexure

**Published:** 1893-03

**Authors:** 


A TREATISE ON DISEASES OF THE RECTUM, ANUS AND SIGMOID FLEXURE, by Joseph M. Mathews, M. D., Professor of Surgery, etc., Kentucky School of Medicine, etc., etc. Published by D. Appleton & Co., N. Y. 1892.

THIS is a book of 537 pages of well written and useful material, both for the student of medicine and the general practitioner. The author claims that he is a pioneer in this class of work, being the first to adopt this special line as a specialty, and the book, therefore, as he states, is largely a compilation of his own individual experience.

In the beginning of the book he gives a table of Differential Diagnosis of the diseases of the Rectum, which is not only interesting, but instructive to any one who is interested in this subject. He also devotes six pages to the importance of Life Insurance Examiners making a thorough examination of the rectum in all cases where there is any suspicion of a diseased condition of those parts, and relates a number of cases where tuberculosis, syphilis and cancer were detected in this way long before any other symptoms were manifest.

Considerable space is given to the chapter on constipation, which the author thinks is second to no other subject in importance to the rectal surgeon. He lays special stress on the action of the external sphincter muscle in this connection.

Chapter IV. is given to the use of antiseptics in rectal surgery, which is of unusual interest at this day and time.

Chapters V. to VIII. is devoted to the subject of Hæmorrhoid.

Chapters VIII. and IX. deals with Fistulo in Ano, and contains a great deal of original thought. Some of his teachings are in direct oposition to the views of the old and some of the more rccent authorities. This refers especially to the advisability of operating on patients who are suffering from lung complications. In this chapter he refers to his *fistulotome*, and points out the character of cases in which its use is indicated as the easiest, simplest and the most satisfactory way of treating this condition.

The chapters on the Nervous or Hysterical Rectum and Neuralgia of the Rectum are of special interest, also the space given to the subject of diseases in the Sigmoid Flexure.

The typographical appearance of the book is excellent, it contains six handsome chromo lithographs showing the operations for hæmorrhoid, fistula in ano, and proplapsus ani, and numerous other illustrations relating to the text.

The book deserves a place in every library where it will, no doubt, prove a valuable addition. W. A. C.

